# Correction to “Investigation of Nickel-Based Reforming Catalysts Under Coking Conditions in a Tailor-Made Test Setup for Performance Evaluation”

**DOI:** 10.1021/acsaenm.6c00028

**Published:** 2026-03-27

**Authors:** Svante P. Ihrig, Michael Wullenkord, Dimitrios Dimitrakis, Christos Agrafiotis, Christian Sattler

In the original version of the paper, Figure 4(a) and (b) were switched. The correct Figure 4 is given below. The conclusions of the work are not affected by the correction of Figure 4.

**Figure 4:**
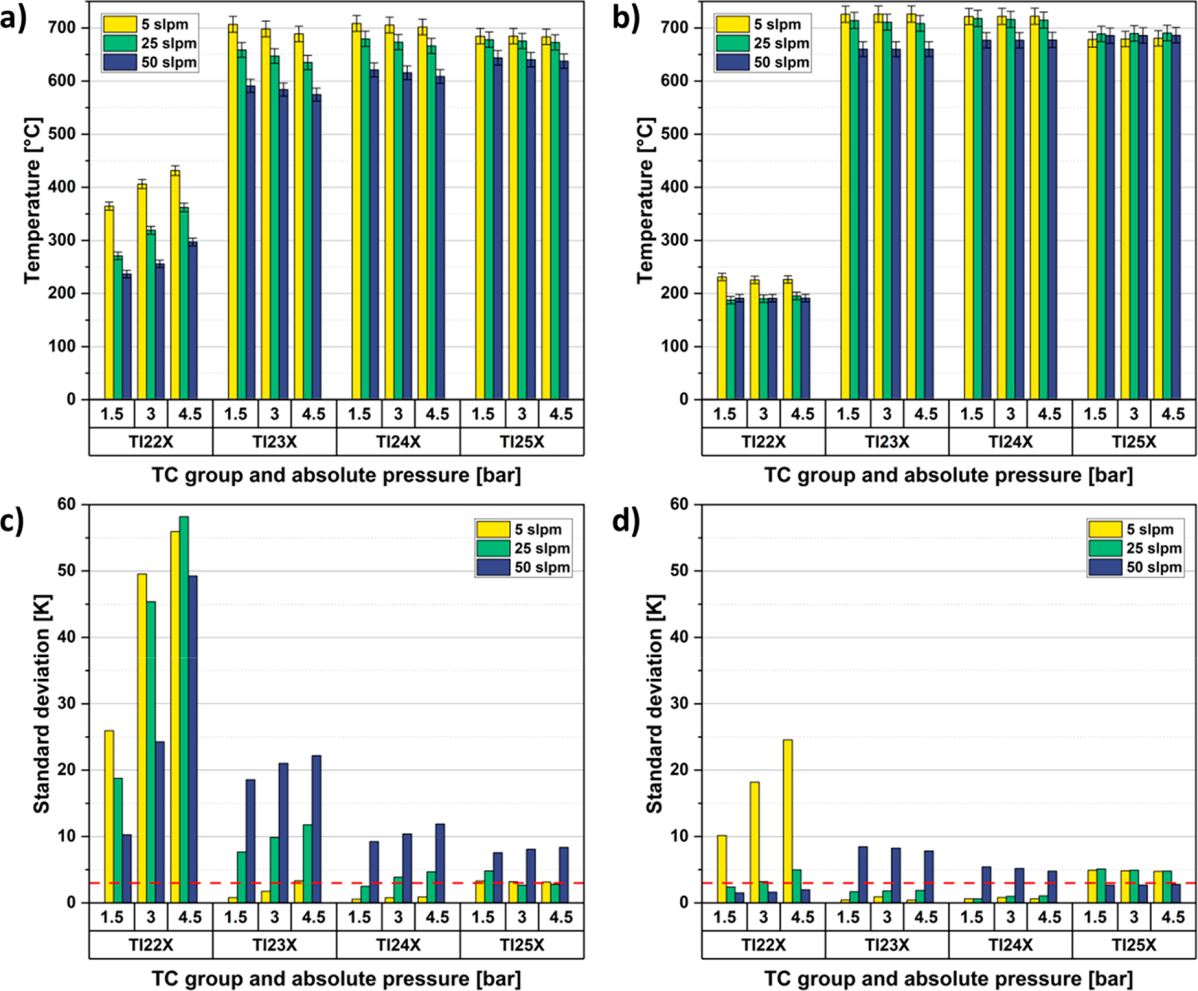
Axial and radial temperature distributions in the reactor cassettes at 725 °C furnace set point, nitrogen flow from 5 to 50 slpm and absolute pressure from 1.5 to 4.5 bar. (a) Average temperatures with empty cassettes. (b) Average temperatures with both cassettes filled with inert material. (c) Standard deviations for (a) with 3σ interval marked as dashed red line. (d) Standard deviations for (b) with 3σ interval marked as dashed red line.

